# Electric Cable Construction Parameter and Its Potential to Foresee the Cable Fire Properties

**DOI:** 10.3390/ma16041689

**Published:** 2023-02-17

**Authors:** Katarzyna Kaczorek-Chrobak, Jadwiga Fangrat

**Affiliations:** Fire Research Department, Instytut Techniki Budowlanej, Filtrowa 1, 00-611 Warszawa, Poland; j.fangrat@itb.pl

**Keywords:** cable parameter, cable fire properties, reaction to fire of cables, fire safety

## Abstract

A cable parameter related to the volume of effective non-combustible content, *Ω*, is proposed, which depends on the ratio of non-metallic, non-combustible component volume to non-metallic, combustible component volume, and the effective area of heat transfer within the cable during the combustion process. The correctness of the proposed cable parameter for circular cables is confirmed by tests and the determination of Spearman’s correlation. High Spearman’s correlation factors (close to −1) were obtained for total heat release and total smoke production as a function of the *Ω* cable parameter. The *Ω* cable parameter might be used in selecting cable samples for large geometric-scale fire testing within the same cable family.

## 1. Introduction

Electric cables are important parts of buildings and any transportation system, such as vehicles, aircraft, and ships. The huge volume of electric cables installed in such objects, and the types of materials used for their production, have a level of environmental impact. They strongly influence fire safety as well. Such fires may present negative safety and environmental impacts, and may exert a strong influence on the external environment and surroundings. Energy conservation and safety needs are contradictory in some situations, and therefore a proper balance between them is necessary [1,2,3].

The fire properties of electrical cables have gained growing interest since they were included in the group of building products in the EU in 2011. There are kilometers of electrical cables installed in each building; therefore, they constitute a significant factor in its fire safety. The large number of cables installed not only strongly increases their fire load but may also facilitate flame spread over a long distance (both horizontal and vertical) in the case of fire [4,5,6] and increase the potential fire toxicity [7]. Cables are an indispensable part of present life, but often pose a potential fire hazard. They provide electricity and signals to various receivers and, based on their end-use application, may be clearly divided into types according to their purpose. Cables are complex objects because they consist of insulation and sheaths made of polymeric materials of various chemical structures, thicknesses, and additives [8].

The use of electric cables has brought to the forefront the need to protect against electric shock, overload and short-circuit current, switching overvoltage, lightning, and numerous heat effects [9], including fire.

The fire properties of electric wires and cables have been extensively studied, both experimentally and theoretically. A valuable review has been presented [10] with the following summary: ‘the complex role of the conductor, specifically whether it is a heat source or heat sink, in the ignition, flame spread, burning, and extinction, has been emphasized throughout this review.’ And ‘a deeper understanding of fire phenomena in real wire and cable is still quite challenging, and attempted inferences for real wire fires based on the qualitative or semi-empirical analysis of limited laboratory data are not yet convincing enough […] there is still a large gap between the fundamental research using laboratory wires and applied research using commercial wires’ [10].

Geometric small- and large-scale tests for measuring heat, smoke, and flame spread have been developed for better understanding the fire behavior of electric cables. These processes have been widely investigated in horizontal conditions, i.e., the cone calorimeter method compared with the large-scale cable test, which shows good reproducibility and repeatability in the case of cable testing (FIPEC project) [11,12].

For these studies, cable families were separated in order to decrease the number of tests for fire classification, according to [13]. Within the same family, as defined in the technical specification [14], cables differed in terms of conductor number and size, but the voltage rating and chemical structure of insulations and sheaths were the same.

The influence of conductors in the form of metallic barriers against flame penetration inside the cable, as well as the volume of inorganics incorporated in the non-metallic elements of cables, such as an outer sheath, bedding, and insulations, were found to be critical in accounting for the fire properties of electrical cables [5,6,7,15].

**Figure 1:**
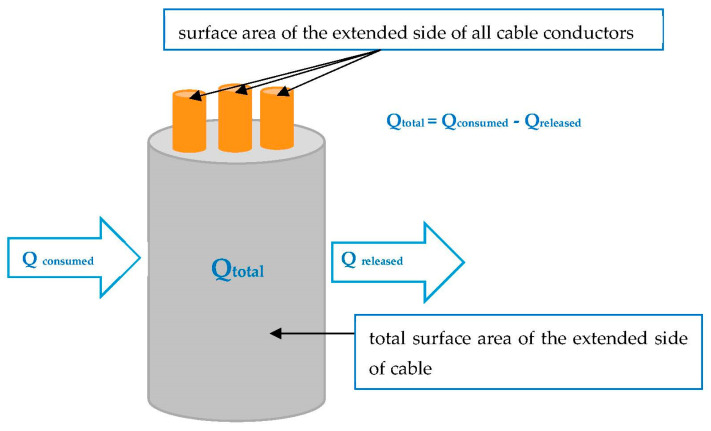
Idealized schematic of heat transfer within the combustion surface area of cables.

In order to explain the physical phenomena associated with cable fire, the conservation of energy law [16] can be interpreted in relation to combustion, which is the rapid oxidation reaction of a substance called fuel, combined with the release of energy in the form of heat. Geometrically, a piece of cable can be considered as a circular or elliptical cylinder. During the combustion processes, the total energy (heat) released is equal to the difference between the heat consumed by the cable and the heat released in the combustion zone (Figure 1). Total heat release is a function of effective combustion surface area [17].

Combustion processes are irreversible, rapid, and complex. They can be classified as physical processes among systems where chemical reactions occur. Total heat release depends on the outer active surface area of the cable and the inner active surface area of the metallic conductors within the total volume of the cable. The cable surface area controls the rate of thermal degradation to release volatile fuel fragments to the combustion zone. For the purpose of this study, a thorough investigation of the polymer combustion process was not needed and has not been performed. However, a good explanation of the polymer combustion process is given in [18]. There, it was stated that “Combustion proceeds by a series of overall exothermic, radical reactions and produces heat and light (flame). Depending on the combustion efficiency, a greater or lesser amount of smoke (particulates, soot) may also be produced”. Moreover, the methods of controlling this combustion process are generally described for both the solid and gas phase.

Based on previous authors’ studies [15,19], it is known that although cable fire performance can be qualitatively well-described by constructional parameters, such as the number of conductors (n), outer diameter (d_cable_), and non-metallic volume per meter of ladder, according to [20] (V_combust_), dependence on fire properties is non-monotonic. This work aimed to elaborate on a new cable parameter which would, more accurately than the currently used χ cable parameter, characterize the cable family with regard to its fire properties. An application-driven study, with the potential of complying with the explicit and implicit concepts, as well as project requirements related to construction, transportation, environmental and safety aspects, were considered.

## 2. Materials and Methods

For the concept of cable parameter, six cables were taken for experiments. The cables with a low number of conductors were used as halogen-free flame-retardant power cables, and cables with more than seven conductors were control cables. Those cables were constructed with the same materials, with the same type of conductor, sheath, and insulation (Table 1).

**Figure 2:**
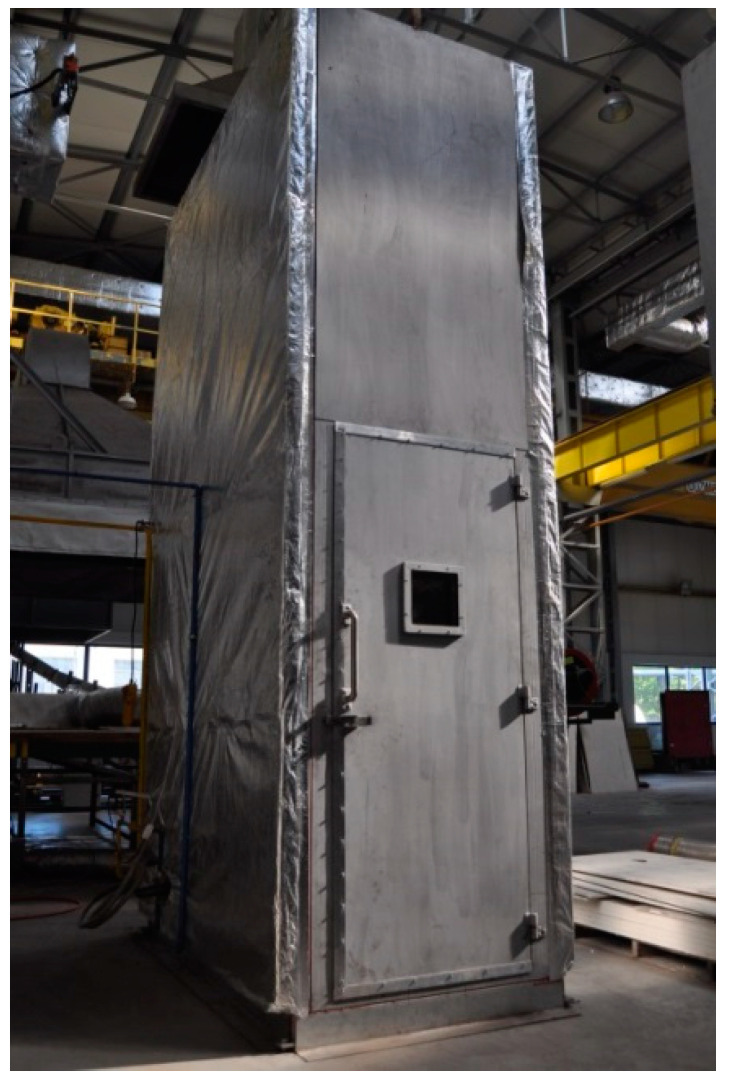
External view test chamber at the accredited ITB Fire Testing Laboratory in Pionki, Poland.

The cable samples (Table 1) were tested experimentally by means of a standard method [20] (Figure 2). This large geometrical test method was chosen to establish the cables’ real-scale configurations (cable trays). Cable samples are tested as a whole product, identical to the ones installed in buildings.

The test apparatus consisted of the following main elements:—regular cuboid chamber with a ventilation system supplemented with the oxygen consumption calorimetry method for determining heat and smoke release rates [11,18,21],—vertical cable specimen tray (ladder) of 3.6 ± 0.1 m (height) × 300 mm (width),—ignition gas burner at the bottom of the tray. A paramagnetic analyzer measured oxygen depletion in combustion effluent in the ventilation duct. Carbon dioxide concentration was measured by means of non-dispersive infrared (NDIR) spectrometers [22]. The test method, therefore, allowed the use of the carbon dioxide generation (CDG) and oxygen consumption (OC) calorimetric methods to obtain a proper assessment of the HRR for materials of unknown composition [23].

**Figure 3:**
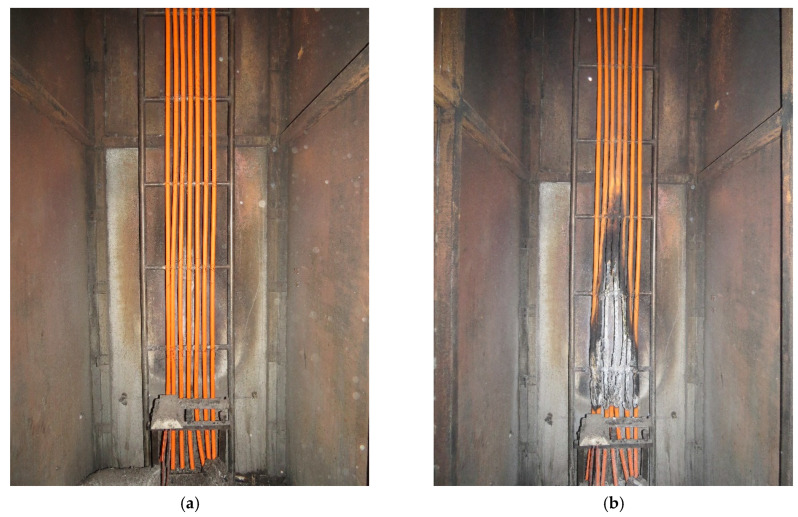
Cable sample No 3 installed on the test ladder: (**a**) before the test, (**b**) after the test.

Cable tray specimens were mounted on a 4 m long ladder (Figure 3a,b) inside the chamber, in order to be tested in their end-use application; depending on the cable parameter, several pieces of each cable were studied during a single test. A nominal HRR level of 20.5 kW, an air flow rate through the chamber of 8000 ± 800 L/min, and a white light detector were used in the burner [20].

For the TGA analysis, the weight of the load material was up to 90 mg, and a heating rate of 50 °C/min up to 1000 °C was used, to simulate the heating rate of real fire [24]. A chemically and thermally neutral alumina-and-platinum pan was used for the transportation of the specimen into a furnace.

## 3. Results of Empirical Tests and Discussion

### 3.1. Cable Parameter Ω Proposal for Circular Cables

The proposed cable parameter, which would be able to foresee the fire properties of electric cables, is a volume of effective non-combustible content *Ω*, which combines all the necessary constructional and material parameters of cables responsible for their fire properties. The *Ω* cable parameter can be calculated using the following Equation (1):

(1) $$\Omega =\frac{1}{z}\left(\frac{V_{combust}}{1+\omega }\right),$$

where:

*Ω*—volume of effective non-combustible content (cable parameter), [l/m of cable]
*z*—surface ratio, [-]
*V_combust_*—non-metallic volume of combustible cable components, l/m bunched cable according to standard [17]
*ω*—non-metallic, non-combustible component volume to non-metallic, combustible component volume ratio, [-]

The surface ratio (*z*) is related to the heat transfer between the active outer surface of the cable and the surface of the metallic conductors inside the cable. Circular cables and circular conductors are circle-based cylinders with known diameters. The surface ratio (*z*) can be defined as the ratio of the surface area of the extended side of all cable conductors to the total surface area of the extended side of the cable (2).

(2) $$z=\frac{\pi hnd_{met}}{\pi hd_{cable}},$$

where:

*d_met_*—diameter of conductor, [m]
*n*—number of conductors, [-]
*d_cable_*—diameter of cable, [m]
*h*—unit length of cable, [m]

### 3.2. Experimental Verification of the Ω Cable Parameter

The non-metallic, non-combustible component volume to non-metallic, combustible component volume ratio (*ω*) can be obtained from the thermogravimetric analysis (TGA) of each separate cable component (outer sheaths, beddings, and insulations) as a sum of the ratios of mass of residue to mass loss of each component. The calculations of each component of Equation (2) for six cables are given in Table 2. The properties of the cables are given in Table 1 and Table 2, and the absolute mass loss Δm, which represents the total mass of non-metallic combustible components of cables, is given in Table 2.

The thesis established above indicated that the total heat release (THR_1200s_) (during the 1200 s period of the fire test) depends on the outer active surface area of the cable and the inner active surface area of the metallic conductors within the total volume of the cable, described by a mathematical Equation (1). Quite a similar relationship occurs in the case of the total smoke production (TSP_1200s_) in the case of cables.

**Figure 4:**
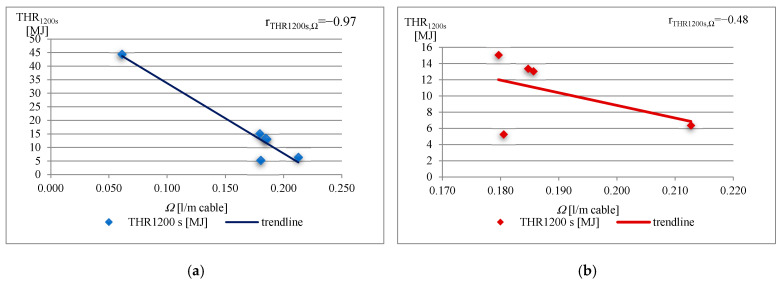
Correlation between THR_1200s_ and *Ω* for the tested cables.

**Figure 5:**
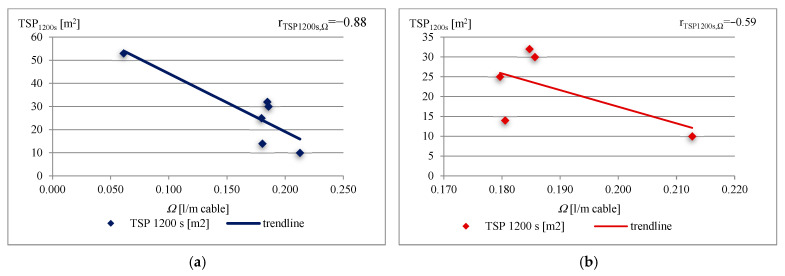
Correlation between TSP_1200s_ and *Ω* for the tested cables.

Spearman’s correlations between THR_1200s_ and the *Ω* cable parameter (Figure 4a), and between TSP_1200s_ and the *Ω* cable parameter (Figure 5a) for cable samples Nos 1, 2, 3, 4, 5, and 6, and the correlation between cable samples except cable sample No 1 (Figure 4b and Figure 5b), have been chosen for showing the relationship between the fire properties and the constructional and material parameters of cables. THR_1200s_ and TSP_1200s_ were obtained during the test, and directly depend on the combustible materials of the cables, which is further discussed.

As mentioned, the THR_1200s_ and TSP_1200s_ strongly depend on combustible material content. Absolute values of Spearman’s correlation factors for both fire properties as a function of the *Ω* cable parameter increase with the number of cables for testing (Figure 4 and Figure 5). However, there is a disturbance in the correlation, in that that the lowest value of the *Ω* cable parameter (*Ω* = 0.061 L/m cable) was obtained for a cable consisting of one copper conductor, without any bedding, and thus a low amount of combustible non-metallic content (sample No 1). These differences in the construction of the one-conductor cable cause a low linear correlation coefficient r (r_THR1200s,*Ω*_ = −0.48 and r_TSP1200s,*Ω*_ = −0.59) (Figure 4b and Figure 5b).

In the case of multi-conductor cables, the lowest value of the *Ω* cable parameter was determined for the three-conductor cable, as expected. The highest value of the *Ω* cable parameter, for cables which consist of 24 conductors rather than 30 conductors, however, highlighted the strong relationship to the combustible non-metallic volume of cables, which is much stronger than the relationship to number of cable conductors.

The values of the linear correlation coefficient r for THR_1200s_ and TSP_1200s_ observed are close to −1 (r_THR1200s,*Ω*_ = −0.97 and r_TSP1200s,*Ω*_ = −0.88) (Figure 4a and Figure 5a), which demonstrates that there is a (negative) linear relationship between them and the volume of effective non-combustible content *Ω*, namely the number of cables, metallic flame barriers (conductor barriers), and combustible non-metallic components of cables.

**Figure 6:**
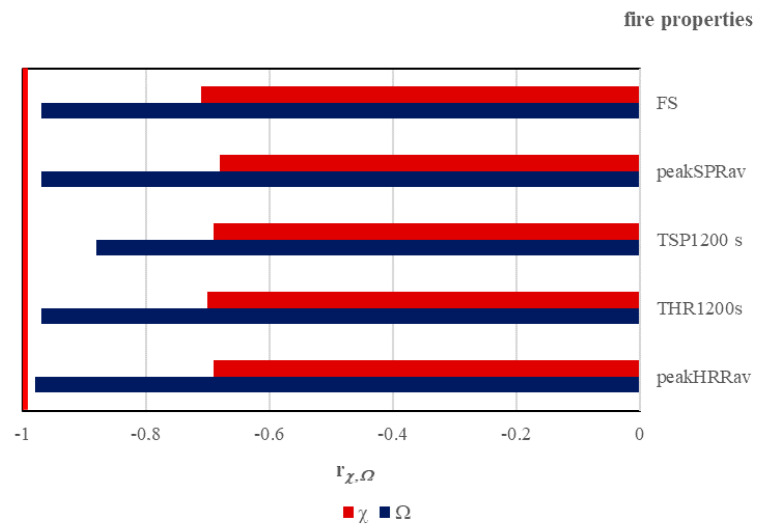
Comparison of *r* values for cable parameters χ and *Ω*.

The previously published research [15] showed that a simple relationship exists between the value of cable parameter *χ* and the number of conductors (*n*) for cables with a number of conductors less than 19. For halogen-free flame-retardant cables with a greater number of conductors, increasing values of heat release rate, total heat release, total smoke production, and smoke release rate were observed, despite the increasing content of the non-metallic volume of cables (*V_combust_*). This means that not only one construction characteristic may be considered when investigating the fire properties of cables. Despite the directly proportional relationship of cable parameter *χ* to the non-metallic volume of cables (*V_combust_*), the same value of *χ* may lead to different fire properties of cables. This forced the need to elaborate on the new cable parameter, which would more accurately characterize the cable family regarding its fire properties. The high correlation factors (Figure 6) show that smoke production and heat release depend significantly on the mass loss of the cable sample, in terms of combustible non-metallic content, which is in line with our explanation of physical phenomena.

It is shown (Figure 6) that the lowest value of the *Ω* cable parameter was obtained for cables without any bedding in the construction, consisting of one copper conductor, thus a low amount of combustible non-metallic content. In the case of multi-conductor cables, the lowest value of the *Ω* cable parameter was determined for the three-conductor cable, as expected. The highest value of the *Ω* cable parameter for cables which consist of 24 conductors rather than 30 conductors, however, highlighted stronger relationship to the combustible non-metallic volume of cables than to the number of conductors inside the cable.

## 4. Cable Parameter Proposal for Non-Circular Cables and Cables with Sector-Shaped Conductors

**Figure 7:**
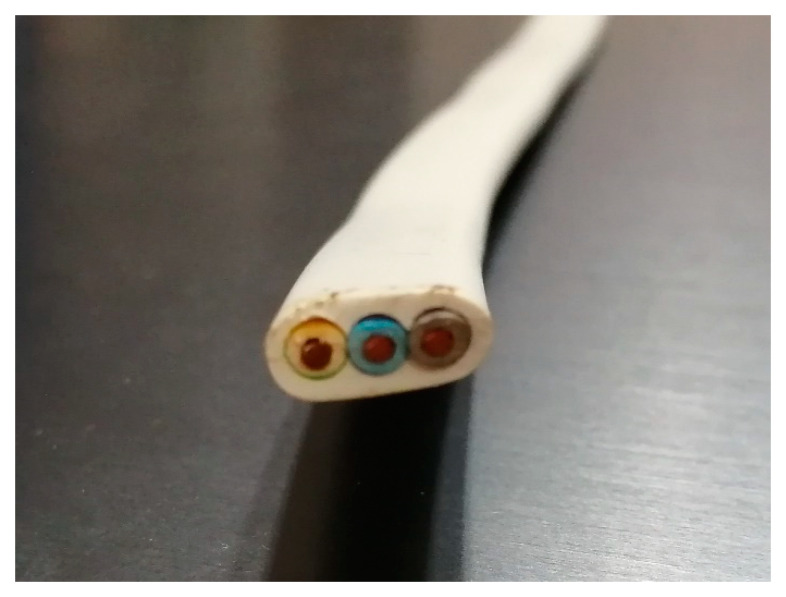
Cross-section of three-conductor non-circular cable.

Analogous dependences are proposed for the *Ω* cable parameter for non-circular cables (Figure 7), which are considered as elliptical cylinders.

The difference between non-circular and circular cables is due to their cross-section, and only factor *z* needs to be derived from a formula. The rest of the components of Equation (1) remain unchanged. Factor *z* depends on the active surface (area of the side surface) that undergoes thermal impact and needs to be calculated using the following Equation (3):

(3) $$z=\frac{\pi hnd_{met}}{S_{cable}},$$

where:

*d_met_*—diameter of conductor, [m]
*n*—number of conductors, [-]
*h*—unit length of cable, [m]
*S_cable_*—area of the side surface of cable, [m^2^]

The area of the side surface of the cable could be calculated using the approximate Equation (4):

(4) $$S_{cable}=\pi h\left(1.5\left(a+b\right)-\sqrt{ab}\right),$$

where:

*h*—unit length of cable, [m]
*a*—major axis, [m]
*b*—minor axis, [m]

**Figure 8:**
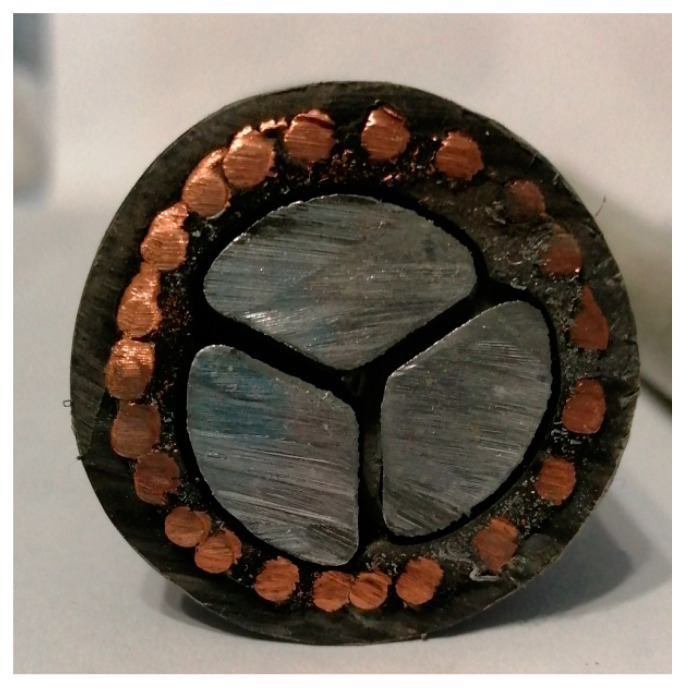
Cross-section of three-conductor sector-shaped cable.

Conversely, sector-shaped conductors in cables (Figure 8) should be treated as prisms with the base of an isosceles triangle, whereas the cable is always circular. This takes place in the case of 3-, 4-, and 5-conductor cables (classes 1 and 2), differing depending on the number of conductors in the cable:

(1) 3-conductor cable: internal angle in an isosceles triangle equal to 120°,
(2) 4-conductor cable: internal angle in an isosceles triangle equal to 90°,
(3) 5-conductor cable: internal angle in an isosceles triangle equal to 72°.

On this basis, the proposed surface ratio *z* could be determined as the ratio of the surface areas of the side of the respective prisms, and calculated using the following Equation (5):

(5) $$z=\frac{nP_{met}}{\pi hd_{cable}},$$

where:

*n*—number of conductors, [-]
*d_cable_*—diameter of cable, [m]
*h*—unit length of cable, [m]
*P_met_*—area of the side of conductor, [m]

The area of the side surface of the single conductor might be determined assuming that the prism’s base is an isosceles triangle (Equation (6)).

(6) $$P_{met}=h\left(2a+b\right)$$

where:

*h*—unit length of cable, [m]
*a*,*b*—sides of the triangles, [m]

In summary, the proposed cable parameter *Ω* is a precise reflection of the fire performance of electric cables. The proposed cable parameter may apply to every construction of electric cables.

## 5. Concluding Remarks

Combustion processes determine the fire behavior, i.e., the fire properties, of electric cables. They are dependent on the constructional and material characteristics of electric cables, as presented in the previous authors’ works. Certain constructional-material parameters of cables increase the mechanical and electrical properties of cables, but at the same time contribute to the deterioration of their fire properties (such as flame spread, heat release, and smoke generation parameters) and, thus, decrease the fire safety of various objects (vehicles, buildings, and transportation and infrastructure systems). Particularly, the geometric and material characteristics of the conductors, as well as the amount of non-metallic combustible materials, are the important factors.

Although the fire properties of cables can be described qualitatively well by constructional parameters such as the number of wires (*n*), the external diameter (*d_cable_*), and the non-metallic volume per meter of cable (*V_combust_*), their dependence on fire properties proved to be non-monotonic in some cases. The main idea of the *Ω* cable parameter is to consider the ratio of the volume of non-metallic components to the volume of non-metallic combustible elements of the cable, and the effective heat transfer area inside the cable, for determining and grouping the fire properties of cables. Significantly high Spearman’s correlation factors (close to −1) were achieved for THR_1200s_ and TSP_1200s_ as a function of the cable parameter *Ω*. Performed experiments on the relationship of fire properties to the design parameters associated with the number of conductors and their shape, selected according to the criteria described above, were supplemented by the theoretical analysis of the *Ω* cable parameter for cables with an elliptical cross-section (non-circular cables) and for cables with sector-shaped conductors determined from the accepted geometry of idealized solids. For non-circular cables, the shape of a cylinder with an elliptical base was selected, and for cables with sector-shaped conductors, wires in the shape of a prism’s base of isosceles triangles were selected.

In summary, the cable parameter *Ω* might be a precise predictive indicator of cable combustibility. It would allow a better selection of cables within the cable family with an increasing number of conductors. The construction of electric cables, especially the amount of non-metallic combustible materials and conductor metal barriers, prompted the modification of cable parameters to include both considered characteristics. The proposed *Ω* cable parameter depends on the volume of effective non-combustible content, and is presented in liters of non-combustible non-metallic components per meter of cable. Its value is a very good indicator, allowing us to foresee electric cables’ fire properties before performing costly real-scale experiments. The *Ω* cable parameter may ease the necessary preparatory work, as well as shorten the time needed for a precise choice of cables. This original, and relatively simple, but efficient, relationship between the constructional-material parameters of electrical cables and their fire behaviour was demonstrated.

## 6. Directions for Further Studies

Expanding research on the *Ω* cable parameter and confirmation of its application potential will be the subject of further work.

Further studies are necessary in order to obtain more data, allowing a better understanding and explanation of the significant influence of electrical installations on flame spread in various objects and systems. They constitute significant risks to the fire safety through increasing the ability of fire development.

## Figures and Tables

**Table 1:** Characteristics of cable samples.

Cable Sample No	1	2	3	4	5	6
Cross-section of cables	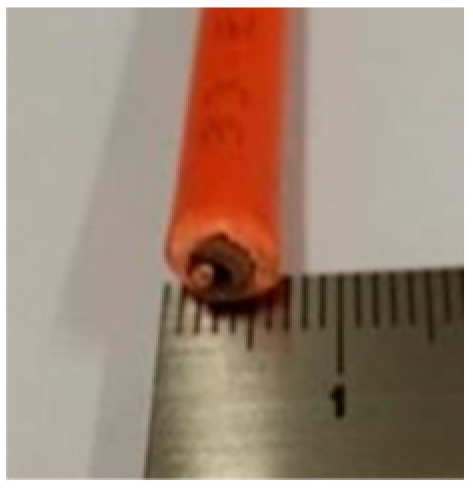	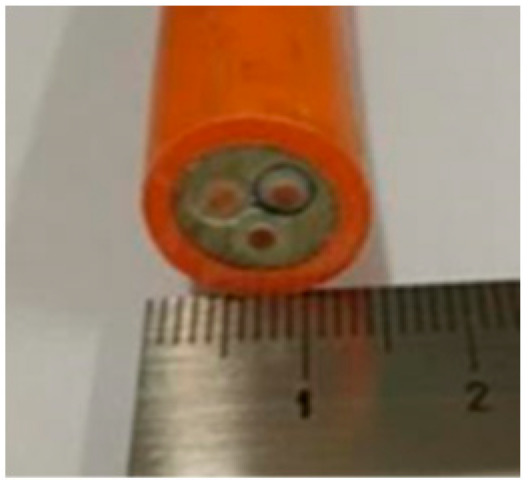	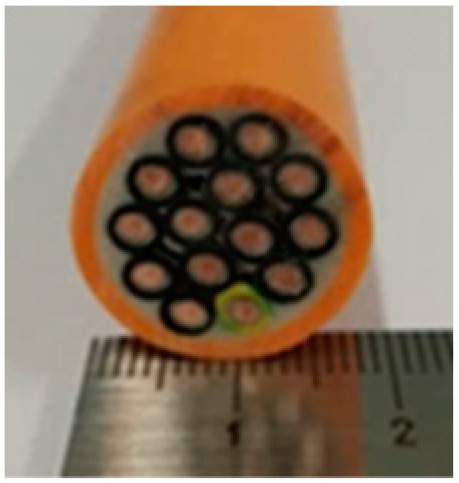	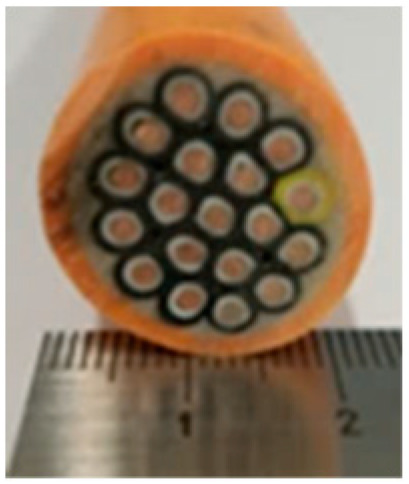	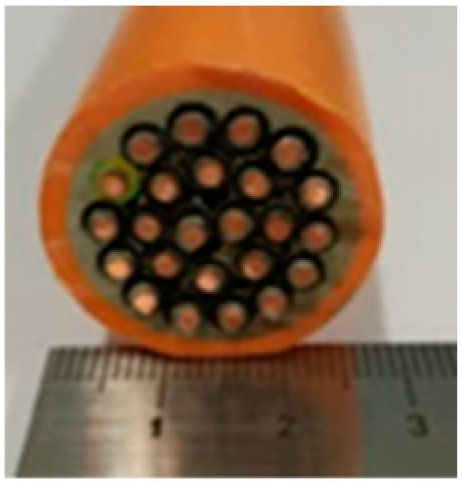	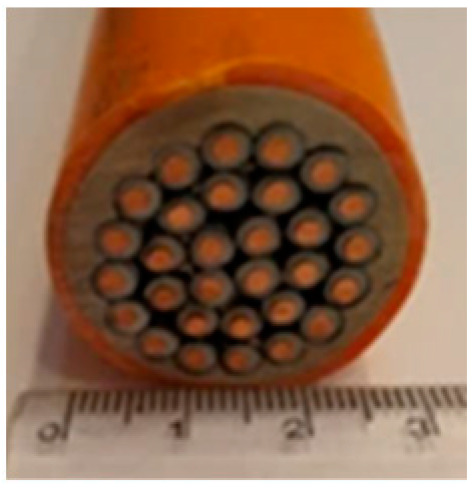
Cable Size	1 × 1.5 mm^2^	3 × 1.5 mm^2^	14 × 1.5 mm^2^	19 × 1.5 mm^2^	24 × 1.5 mm^2^	30 × 1.5 mm^2^
d_cable_, mm	6.7	13.6	22.4	24.7	27.0	30.5
Conductors	Copper, circular					
Insulations	Silane cross-linked polyolefin (XLPO) and mica tape					
Bedding	none	Flame retardant cross-linked polyethylene (XLPE)				
Outer Sheath	ethylene/vinyl/acetate (EVA) copolymer filled with aluminium trihydrate (ATH) and zinc borate (ZnB) as a flame retardant					

**Table 2:** Calculation of the *Ω* cable parameter for tested cable samples.

Cable Sample No	*n*, [-]	*d_met_*, [m·10^−3^]	*d_cable_*, [m·10^−3^]	*V_combust_*, [l/m Cable]	Δm ^*^, [-]	*ω*, [-]	*z*, [-]	*Ω*, [l/m Cable]
1	1	1.38	6.7	0.032	0.39	1.54	0.206	0.061
2	3	1.38	13.6	0.126	0.45	1.24	0.304	0.185
3	14	1.38	22.4	0.315	0.49	1.03	0.863	0.180
4	19	1.38	24.7	0.387	0.50	1.02	1.062	0.181
5	24	1.38	27	0.492	0.53	0.89	1.227	0.213
6	30	1.38	30.5	0.562	0.45	1.23	1.357	0.186

## Data Availability

Not applicable.
